# Layered Double Hydroxides for Photo(electro)catalytic Applications: A Mini Review

**DOI:** 10.3390/nano12193525

**Published:** 2022-10-09

**Authors:** Cheng Li, Huihua Jing, Zhong Wu, Denghui Jiang

**Affiliations:** 1 School of Physics and Electronic Sciences, Changsha University of Science and Technology, Changsha 410114, China; 2 Hunan Provincial Institute of Product and Goods Quality Inspection, Changsha 410116, China; 3 Tianjin Key Laboratory of Composite and Functional Materials, Key Laboratory of Advanced Ceramics and Machining Technology (Ministry of Education), School of Materials Science and Engineering, Tianjin University, Tianjin 300072, China; 4 Hunan Provincial Key Laboratory of Flexible Electronic Materials Genome Engineering, Changsha University of Science and Technology, Changsha 410114, China; 5 Hunan Province Higher Education Key Laboratory of Modeling and Monitoring on the Near-Earth Electromagnetic Environments, Changsha University of Science and Technology, Changsha 410114, China

**Keywords:** LDHs, two-dimensional layered materials, photochemistry, structure-performance correlations

## Abstract

Chemical energy conversion strategies by photocatalysis and electrocatalysis are promising approaches to alleviating our energy shortages and environmental issues. Due to the 2D layer structure, adjustable composition, unique thermal decomposition and memory properties, abundant surface hydroxyl, and low cost, layered double hydroxides (LDHs) have attracted extensive attention in electrocatalysis, photocatalysis, and photoelectrocatalysis. This review summarizes the main structural characteristics of LDHs, including tunable composition, thermal decomposition and memory properties, delaminated layer, and surface hydroxyl. Next, the influences of the structural characteristics on the photo(electro)catalytic process are briefly introduced to understand the structure–performance correlations of LDHs materials. Recent progress and advances of LDHs in photocatalysis and photoelectrocatalysis applications are summarized. Finally, the challenges and future development of LDHs are prospected from the aspect of structural design and exploring structure-activity relationships in the photo(electro)catalysis applications.

## 1. Introduce

Globalization and industrialization development have accelerated population increase and economic development, which greatly increase demand of fossil fuels [1]. As of 2018, fossil energy still accounted for 80% of the world’s primary energy [2]. The huge consumption of fossil energy brings numerous ecological and social problems, such as the greenhouse effect [3,4], environmental pollution [5,6], and reduction of fossil energy [7,8,9]. Solar and hydrogen energy attract attention as promising green and clean energy sources to address our energy shortages and environmental problems. However, it is necessary to convert solar energy and excess electrical energy into chemical energy stored in chemical molecules, due to limits of time and space [10]. Among various chemical energy conversion strategies, photocatalysis and electrocatalysis are attractive approaches for converting solar energy and produce hydrogen or hydrocarbon fuels, such as water splitting and CO_2_ reduction by photo(electro)catalysis [11,12,13,14,15,16]. A variety of materials have been exploited in photo(electro)catalytic energy conversion. Among these materials, two-dimensional (2D) materials have attracted tremendous interest due to its high charge mobility and large specific surface area [17,18,19].

Layered double hydroxide (LDH) is a classical 2D layered material. Natural LDH (hydrotalcite) was discovered in 1842 [20]. In 1942, Feitknecht and Gerber first synthesized LDHs and proposed the concept of a double sheet structure [20,21]. Until 1969, the lamellar structure of LDHs was determined by Allmann and Taylor using single crystal X-ray diffraction [22]. Due to the unique structures and properties, the interest in LDHs is gradually increasing and the application is also widely explored [23,24,25,26,27,28]. For example, the interlayer space, exchangeable guest anions, and high specific surface areas are very beneficial for the removal of pollutants (organic contaminant, heavy metal ions, etc.) and the loading of drugs, resulting in potential applications in the fields of environmental protection and drug delivery [24,26]. Due to the 2D layer structure, adjustable composition, high specific surface area and abundant active sites, and mass producibility, LDHs have attracted extensive attention in electrocatalysis [29,30,31,32], photocatalysis [33,34,35], and photoelectrocatalysis [36,37,38]. Among the many structural features of LDHs, tunable structure, such as controllable composition (tunable metal cations and guest anions) and size (delamination of LDH), are huge advantages for function-oriented design of LDHs in photo(electro)catalysis. Thus, it is crucial to understand the relationship between structure and photo(electro)catalytic performance of LDHs.

In this review, we briefly highlight the influences of the intrinsic structural characteristics of LDHs on the photo(electro)catalytic process to better understand the structure–performance correlations. Recent progress and advances of LDHs in photocatalysis and photoelectrocatalysis applications (water splitting, CO_2_ reduction, and contaminant degradation) are summarized in Section 2. Finally, the challenges and future development of LDHs are also examined from the aspect of structural design and exploration of structure–activity relationships in photo(electro)catalysis applications.

## 2. Structures and Properties of LDH

**Figure 1:**
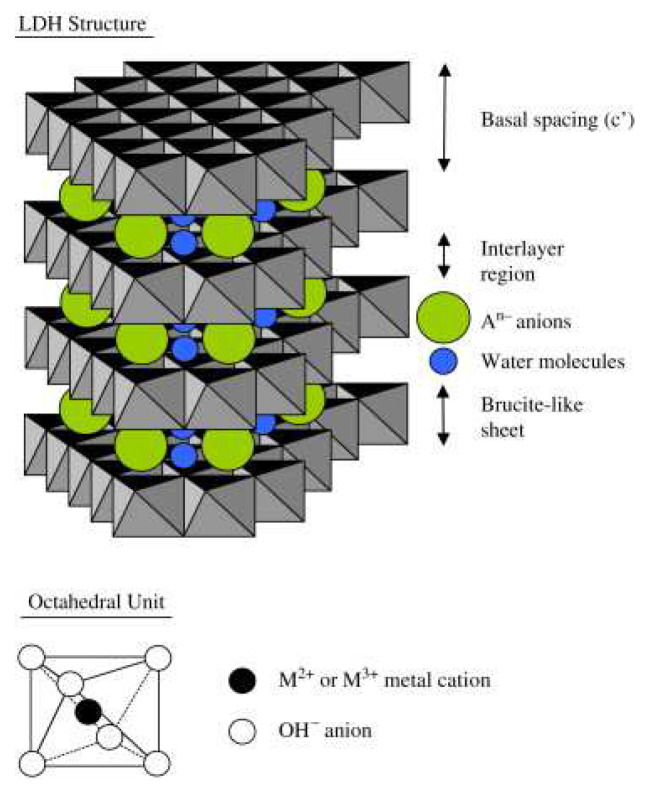
Structural model of LDH [40]. Copyright 2007 Elsevier.

LDHs, also called hydrotalcite-like compounds, are two-dimensional layered clays. LDHs consist of host layers with metal cations and interlayer anions to keep charge balance with H_2_O molecules. Thus, the general formula for LDHs is written as [M^2+^_1−x_M^3+^_x_(OH)_2_]^x+^[A^p−^_x/p_]^x−^·mH_2_O, where M^2+^ is divalent metal cation (e.g., Fe^2+^, Mg^2+^, Ni^2+^, Co^2+^, Cu^2+^, Mn^2+^, Zn^2+^, Cd^2+^, Pd^2+^, and Ca^2+^), M^3+^ is trivalent metal cation (e.g., Co^3+^, Al^3+^, Mn^3+^, Fe^3+^, Cr^3+^, Ga^3+^, V^3+^, and Tb^3+^), X = M^3+^/(M^2+^+M^3+^), (0.2 ≤ x ≤ 0.33), A^p-^ is an interlayer anion, and m represents the number of H_2_O molecules [2,39,40], as shown in Figure 1. On occasion, there are metal cations of M^+^ and M^4+^ in LDHs, but only with the exception of M^+^ being Li^+^ and M^4+^ being Ti^4 +^ [18]. The main structural properties of LDH are tunable composition, thermal decomposition, memory properties, delaminated layer, and abundant surface hydroxyls [17]. Next, we will elaborate on the effect of these structural properties on photo(electro)catalysis performance.

### 2.1. Adjustable Composition

The most significant structural property of LDH is the compositional flexibility, including tunable metal cations in the host layer and guest anions in the interlayer. The tunability of composition significantly affects the physicochemical properties of LDH. We will discuss the influences of tunable composition on the physicochemical properties and photoelectrocatalytic performance of LDH.

#### 2.1.1. Regulating Energy Band Structure

**Figure 2:**
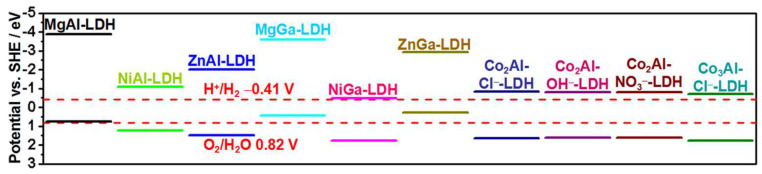
The position of M^II^M^III^−LDHs band edge [41]. Copyright 2015 American Chemical Society.

The varied metal cation species and ratios modulate the composition of LDHs, and their physicochemical properties change significantly. The band structure of the LDH is usually regulated by the changed types and ratios of metal cations in the host layer, which change the range of light absorption and oxidation–reduction potential of LDH. Xu et al. [41] found band gaps of Mg and Zn-based LDH were greater than 3.1 eV, whereas the Co and Ni-based LDH samples absorbed visible light with a band gap lower than 3.1 eV (Figure 2). Guo et al. [42] loaded TiO_2_ to three different cobalt-based LDHs (CoAl-LDH, CoCr-LDH, and CoFe-LDH). The Ti-TiO_2_@CoCr-LDH had the optimal photoelectrocatalytic (PEC) performance with a 43% increase in photocurrent in those samples. This is because the band structure of CoCr-LDH has the best matching with reduced TiO_2_, resulting in the best water oxidation performance.

The changed ratio of metal cations can also adjust the band gap and light absorption of LDHs. Han and Yang et al. [43] reported that BiVO_4_/NiFe-LDH core/shell heterostructure films had four times higher photocurrent intensity than that of pure BiVO_4_ at 1.23V vs. reversible hydrogen electrode (RHE). The higher content of Fe^3+^ in NiFe-LDH resulted in a smaller band gap and stronger light absorbance and conductivity. Parida et al. [44] fabricated the ternary series of Mg/Al + Fe-CO_3_ LDHs by adjusting the rate of Al/Fe. The Fe^3+^ doping increased the visible-light absorption of MgAl-LDHs, resulting in the better H_2_ evolution performance.

#### 2.1.2. Promoting Electron-Hole Pairs Separation

**Figure 3:**
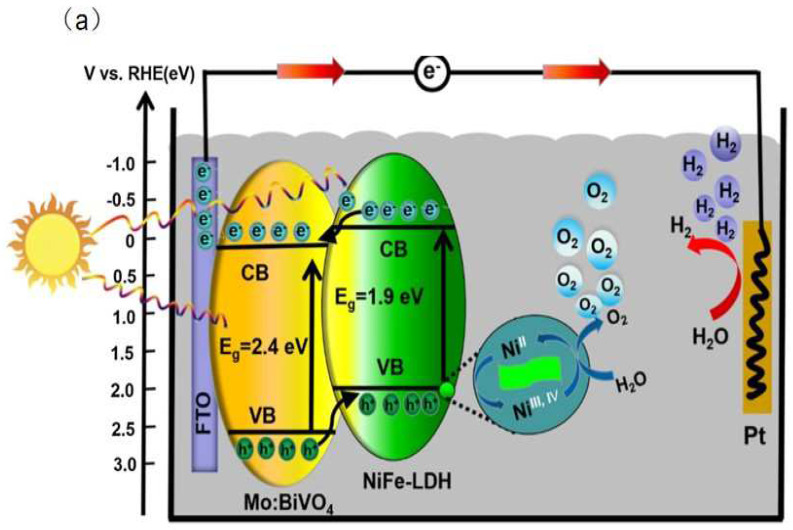

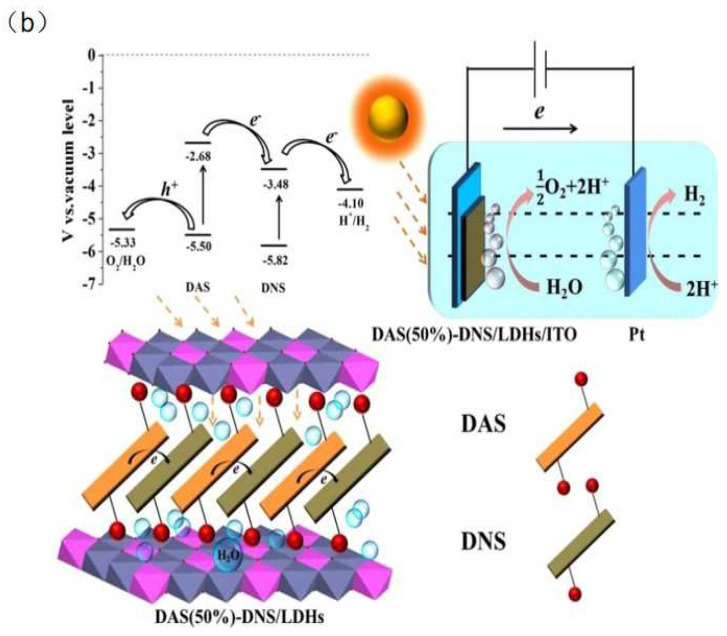
(**a**) PEC water splitting mechanism using the NiFe−LDH/Mo−BiVO_4_ heterostructure photoanode [45]. Copyright 2018 Elsevier. (**b**) The possible photo−generated charges transfer mechanism of DAS (50%)−DNS/LDHs photoanode [47]. Copyright 2015 Springer Nature.

The variable valence state of metal cations of LDHs directly promotes the transfer and separation of charge carriers. Low-valence metal cations are oxidized to high-valence metal cations by the photogenerated holes, which improve the transfer and separation of photogenerated charge carriers. For example, Bai et al. [45] synthesized the NiFe-LDH/Mo-BiVO_4_ heterostructure by an electrodeposition method. The photogenerated holes transferred from BiVO_4_ nanoparticles to NiFe-LDH due to a type II staggered band structure of the heterostructures. At the same time, the photogenerated holes oxidized Ni^2+^ from NiFe-LDH to Ni^3+^and Ni^4+^. The Ni^3+^and Ni^4+^ take part in oxygen evolution reaction (OER) and improve the performance of PEC for decomposing water (Figure 3a). In the work of Shao et al. [14], a ZnO@CoNi–LDH core−shell nanoarray was prepared by an electrosynthesis method. The Co^2+^ was oxidized to Co^3+^/Co^4+^ by the photogenerated holes, which enhanced the efficiency of photogenerated charge carrier separation. Moreover, the Co^3+^/Co^4+^ served as co-catalysts to improve water splitting ability.

Suitable interlayer anions facilitate the transport and separation of charge carriers. Hunter et al. [46] synthesized different interlayer anions inserted into NiFe-LDH samples. The experimental results indicated that all interlayer anions were replaced by CO_2_ in the air to CO_3_^2−^, which had the highest catalytic activity. In non-CO_3_^2−^ interlayer anions, the catalytic activity is a function of the alkalinity of the interlayer anion. The interlayer anions with more negative charges act as stronger proton acceptors and electron donors than interlayer anions with one negative charge. Zheng et al. [47] obtained 4,4-diaminostilbene-2,2-disulfonate (DAS) and 4,4-dinitro-stilbene-2,2-disulfonate (DNS) co-intercalated Zn_2_Al-LDH nanosheets. Due to the matched HOMO/LOMO energy levels of DAS and DNS, the photogenerated electrons of DAS efficiently migrate to DNS under UV-visible-light illumination (Figure 3b). When the percentage of DAS is 50%, the DAS (50%)-DNS/LDHs exhibit excellent photogenerated charge separation ability and stability. Photogenerated electron transfer within the interlayer anion was achieved with water splitting.

#### 2.1.3. Adjusting Selectivity of Reactions

The different types of metal cations of LDHs lead to different active sites of the reaction and thus different products. The different positions of the conduction bands of the photocatalysts determine the different reduction capabilities, leading to the different selectivity in photo(electro)catalytic reactions [48]. Xiong et al. [49] prepared a series of Zn-based layered ZnM-LDH (M = Ti^4+^, Fe^3+^, Co^3+^, Ga^3+^, Al^3+^) by a co-precipitation method. The varied M^3+^ or M^4+^ in the ZnM-LDH could precisely adjust the product selectivity of the CO_2_ reduction. The experimental and computational results revealed that d-band center positions of the metal cations dominated the adsorption strength of CO_2_ and, ultimately, product selectivity. The d-band centers of intralayer metal ions of ZnTi-LDH, ZnGa-LDH, and ZnAl-LDH were relatively adjacent to the Fermi level, which facilitated the reduction of CO_2_ to CH_4_ (ZnTi-LDH) and CO (ZnGa-LDH and ZnAl-LDH). ZnFe-LDH and ZnCo-LDH cannot reduce CO_2_ but induce water desorption and hydrogenation due to the d-band centers of Fe^3+^ and Co^3+^ further away from the Fermi level. Zhao et al. [50] investigated the electronic properties, reaction path, and reaction kinetics of CO_2_PR in 10 M^II^_2_M^III/IV^-NO_3_-LDHs (M^II^ = Mg^2+^, Co^2+^, Ni^2+^, Zn^2+^; M^III^ = Al^3+^, In^3+^, Cr^3+^, Fe^3+^; M^IV^ = Ti^4+^) by Hubbard-corrected density functional theory. The calculation showed that all LDHs might exhibit CO_2_PR except Ni_2_Al-LDH and Ni_2_FeNO_3_-LDH. Among the remaining eight LDHs, the favorable products of the others were CH_4_, except for the product of Co_2_Fe-NO_3_LDH, which was HCOOH. According to the relationship between the effective driving force (ΔΔGb) of CO_2_ reduction to CH_4_ or CO and the adsorption energy of CO_2_, which resembled the relationship between ΔΔGb and valence band maximum (VBM) of LDH, Mg_2_In-LDH was most likely to photocatalytically reduce CO_2_ to CH_4_, whereas Mg_2_Al-NO_3_-LDH was most likely to reduce CO_2_ to CO.

#### 2.1.4. Improving Absorption Capacity

LDHs with exchangeable interlayer anions are widely used to adsorb harmful anions or contaminants of wastewater and polluting soil. The type of interlayer anion affects the adsorption capacity of LDH for the anions in solution. HONGO et al. [51] prepared MgAl-LDH with Cl^−^, ${\mathrm{NO}}_{3}^{-}$, or ${\mathrm{SO}}_{4}^{2-}$ as the interlayer anion to adsorb harmful anions (F^−^, ${\mathrm{CrO}}_{4}^{2-}$, ${\mathrm{HAsO}}_{4}^{2-}$, and ${\mathrm{HSeO}}_{3}^{-}$) using a co-precipitation method. The LDHs with different interlayer anions showed excellent attraction for harmful anions and display adsorption capacity in the order ${\mathrm{NO}}_{3}^{-}$ > Cl^−^ > ${\mathrm{SO}}_{4}^{2-}$. They concluded that the two adsorption mechanisms of LDH for anions were fast adsorption on the surface and slow interlayer anion exchange. The exchange rate of interlayer anions depends on the strength of the interaction between interlayer anions and LDH. The stronger the interaction of interlayer anion and LDH, the weaker the ion exchange capacity of the LDH, resulting in a poorer adsorption capacity of anion. In the first minute, the fast adsorption process is caused by the synergy of two adsorption mechanisms. However, the surface anion adsorption by anion exchange is usually relatively slow. Based on the experimental results and analysis, they believe that the nanocrystallization and highly Al substituted phase of NO_3_-formed Mg-Al LDH obviously improve the anion adsorption ability, resulting in fast surface adsorption. The fast surface adsorption dominates the adsorption ability for NO_3_-formed Mg-Al LDH.

The interaction of interlayer anion and metal ions dominates the adsorption capacity of LDH and selectivity for different metal ions. Jawad et al. [52] synthesized ${\mathrm{MoS}}_{4}^{2-}$ intercalated FeMgAl-LDH as an absorber to removal heavy metals. The results showed the following order of selection for adsorption: Hg^2+^ ∼ Ag^+^ > Pb^2+^ > Cu^2+^ > Cr^6+^ > As^3+^ > Ni^2+^ ∼ Zn^2+^ ∼ Co^2+^. The adsorbed metal cations can form coordination complexes in the interlayer channels. At the same time, the layered structure of LDH provide a protective space for Fe-MoS_4_ to prevent its oxidation. The adsorption capacity of samples for metal ions was determined by the strength of soft-soft acid base bonding interactions between Fe-MoS and metal ions.

### 2.2. Thermal Decomposition and Memory Property

The calcination process causes significant change of the structure and properties of LDHs. The thermal decomposition process of LDHs generally includes three stages [17]: First, the calcination temperature is below 300 °C, adsorbed water of the interlayer and surface is removed, and the layer structure of LDHs is well maintained. Second, during the calcination process at 300–450 °C, the intralayer hydroxyl groups and water are gradually removed. Third, when the calcination temperature is above 450 °C, the layer structure of LDHs gradually collapses, and a composite oxide (M^2+^M^3+^O) is formed.

Calcined LDHs decompose into complex metal oxides and thus form in-situ heterojunctions between the metal oxides, resulting in improved photo/electrocatalytic performance. Suárez-Quezada et al. [53] synthesized a series of ZnAl-LDH samples calcined at different temperatures. They found that Zn was present as hexagonal ZnO in all samples, and Al was present as ZnAl_2_O_4_ and Zn_6_Al_2_O_9_ depending on calcination temperature. Both ZnAl_2_O_4_ and Zn_6_Al_2_O_9_ can form heterojunctions with ZnO. As the temperature increased, the higher crystallinity led to higher hydrogen production efficiency, reaching a peak at 600 ℃. Mostafa et al. [54] prepared novel 1D CoBiTi-LDH with a bandgap of 2.4 eV and 2D CoBiTi layered double oxides (LDO) with high infrared (IR)-responsivity. After drying at 150 °C for 1D CoBiTi-LDH, 1D CoBiTi-LDH and in-situ formed 2D CoBiTi-LDO formed a novel 3D-heterojunction. The hydrogen evolution reaction (HER) of CoBiTi-LDH/CoBiTiO heterojunction increased nearly four times (∼1255 μmolg^−1^h^−1^) compared with the 1D CoBiTi-LDH. The increased HER of the heterojunction was attributed to the enhancement of light absorbance in the IR-region (53% of sunlight) and the trapping of photoexcited species by the functional groups of CBT-LDH.

The calcined LDHs have a stronger adsorption capacity for anionic dyes than the pristine LDHs due to higher specific surface areas and better reconstruction ability [17]. Li et al. [55] prepared hierarchical ZnAl-LDH by ZnAl-LDOs reaction with carbonate solution. The adsorption capacity of ZnAl-LDHfor methyl orange (MO) is far less than that of LDOs due to the decreased specific surface area, adsorption sites, or positive surface charge. Kim et al. [56] reported that calcination process of MgAl-LDHs induced the crystal deformation and formation of an interlayer structure of layered double oxides, leading to the development of mesopores and increased specific surface area. When LDHs were calcined at 500 °C for 10 h and transformed to LDOs, the specific surface area of LDHs obtained by hydrothermal reaction for 1 day (H1-LDH) and 3 days (H3-LDH) increased from 18.4 and 11.3 m^2^/g to 206 and 187 m^2^/g, respectively. The enhanced specific surface area originated from the developed mesopores of the LDO and larger pore volume.

Interestingly, at a certain temperature, the disordered lamellar structure of calcinated LDHs are restored to its original layer structure by immersing it in water or an aqueous solution containing anions [57]. This unique property of LDH is known as the “memory effect”. Peng et al. [58] obtained MgAl-LDH by intercalating 5-Fluorouracil anions using the memory effect of the LDH. The as-prepared samples not only showed improved corrosion resistance, but also inhibited human bile duct cancer cells. Thus, the intercalation of anions in interlayer by the memory effect of LDH is an efficient approach for designing functionalized LDHs [18]. However, some LDHs, such as Ni–Cr, Ca–Al, and Co–Al, have irreversible thermal decomposition behavior, and their lamellar structure cannot be recovered [59].

### 2.3. Delamination of LDH

The LDHs possess a typical layered structure whose layers are connected by strong interlayer electrostatic interactions and interlamellar hydrogen bonding [18]. Although delamination of LDHs remains a big challenge (especially for monolayer LDH) [60], delamination is still an attractive way to improve photo(electro)chemical activity and expand the applications for LDH nanomaterials. This is because the delaminated LDHs have a larger specific surface area, more active sites, and higher electron transport efficiency. Hu et al. [61] delaminated CoCo-LDH, NiCo-LDH, and NiFe-LDH using a liquid phase exfoliation method. The delaminated nanosheets have lower overpotential. When η = 300 mV, the current densities of the CoCo-LDH, NiCo-LDH, and NiFe-LDH nanosheets were 2.6, 3.4, and 4.5 times that of their bulk LDHs, respectively.

Delamination of LDHs introduces more vacancy defects and thus increases number of reactive sites [19]. For example, Wang et al. [62] prepared ultrathin CoFe LDH nanosheets by exfoliation of bulk CoFe LDHs with nitrogen plasma. The exfoliation process induces formation of defects of ultrathin CoFe LDHs nanosheets. The defects increase the dangling bonds near reactive sites and decrease the coordination number of reactive sites, resulting in the improved electrocatalytic activity.

### 2.4. Hydroxyl Groups on the LDH Surface

**Figure 4:**
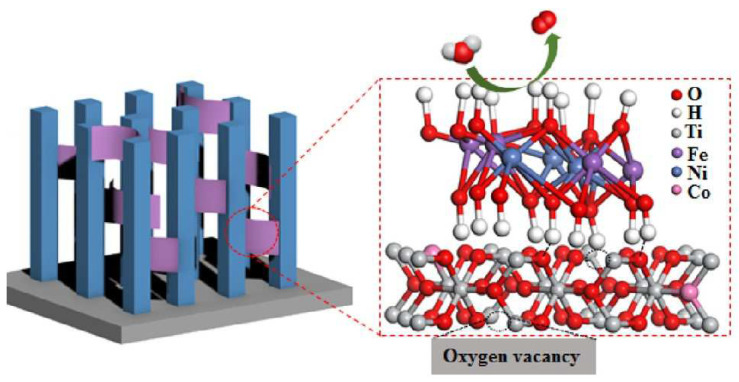
The hydrogen bonds formed between NiFe-LDH and Co-TiO_2_ [13]. Copyright 2020 American Chemical Society.

The LDH surface has abundant surface hydroxyl groups that are nearly perpendicular to the host layer [18]. The hydroxyl groups not only effectively adsorb reactants [63], but also form interfacial chemical bonds with other semiconductor surfaces, thereby facilitating the transport of interfacial charge carriers [15]. For example, Liu et al. [13] deposited NiFe-LDH onto Co-intercalated TiO_2_ by electrodeposition. The hydroxyl groups of NiFe-LDH form hydrogen bonds with TiO_2_ (Figure 4). Therefore, under illumination, the holes in Co-TiO_2_ VB can be transferred to the VB of NiFe-LDH through hydrogen bonding in time to participate in water decomposition, which improves transfer and separation of interfacial photogenerated charge.

## 3. The Photo(Electro)Chemical Applications of LDHs

### 3.1. Water Splitting

When the energy of light harvesting is more than the bandgap energy of LDHs, the electrons in the valence band of LDHs would inject into the conduction band and leave photogenerated holes in the valence band. The photogenerated electrons and holes will migrate to LDH surfaces to participate in a hydrogen evolution reaction and an oxygen evolution reaction. However, in photoelectrocatalysis, the photogenerated electrons will drift to the cathode to participate in a hydrogen evolution reaction, whereas the photogenerated holes will drift to the anode to participate in an oxygen evolution reaction.

Water splitting by photochemistry can be divided into three steps [17]: (i) water adsorption. LDHs and LDH compounds will directly contact water without a concentration gradient. The ability of water adsorption is determined by the specific surface area of LDHs. (ii) Separation and migration of charge carriers: High separation and migration rate of carriers greatly improve the performances of LDH water splitting by photochemistry [64]. (iii) Surface redox reaction: The valence band maximum should be greater than the potential of O_2_/H_2_O (1.23 V vs. normal hydrogen electrode (NHE)), and the conduction minimum should be less than the potential of H^+^/H_2_ (0 V vs. NHE) [65].

Hydrogen is an excellent clean energy and has many potential applications [66], such as hydrogen electric vehicles [67], reduction iron in industry [68], treatment in clinical applications [69], and so on. Water splitting by photochemistry is one effective method to evolve hydrogen. However, the high charge carrier recombination and the low efficiency of hydrogen evolution limit commercial scale production. Among many photo/ electrocatalysts, LDHs have attracted wide attention in photo(electro)catalytic water splitting, due to high specific surface areas, highly dispersed metal active sites, adjustable composition, and low cost [17]. However, many drawbacks of LDHs, such as low conductivity, low carrier mobility, and high electron-hole recombination rate, greatly hinder the photo(electro)catalytic applications [17,70]. Thus, many modification methods of LDHs have been used to improve the photo(electro)catalytic performance.

**Figure 5:**
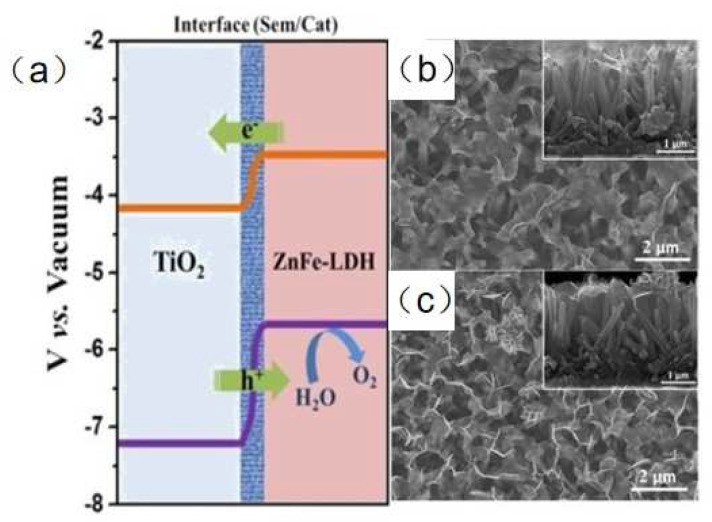
(**a**) The band structure of TiO_2_/ZnFe−LDH heterostructure. SEM images of (**b**) TiO_2_/ZnFe−LDH-E and (**c**) TiO_2_/ZnFe−LDH-PE NAs [72]. Copyright 2017 Elsevier.

Constructing LDH-based heterostructure is an effective strategy to enhance the photochemical hydrogen evolution performance for LDHs. Chen et al. [71] successfully prepared hierarchical CoNi-LDH modified TiO_2_ nanotube arrays (NTAs) by a quick electrochemical deposition method. The photocurrent density of TiO_2_@CoNi-LDH NTAs was 4.4 mA·cm^−2^ (vs. RHE), which was 3.3 times higher than that of pure TiO_2_. The band gap of TiO_2_@CoNi-LDH NTAs was smaller than that of pristine TiO_2_. When light radiation is introduced during the synthesis of the heterostructure, the interface of heterostructure become more compact, leading to better separation ability of charge carriers. Zhang et al. [72] obtained two types of ZnFe-LDH/TiO_2_ nanoarrys (NAs) by photo-assisted electrodeposition (TiO_2_/ZnFe-LDH-PE) method and electrochemical deposition method (TiO_2_/ZnFe-LDH-E), respectively. The photocurrent density of TiO_2_/ZnFe-LDH-PE was 2.29 and 1.31 times than that of pure TiO_2_ and TiO_2_/ZnFe-LDH-E, respectively. For pristine TiO_2_, the interface formed between TiO_2_ and ZnFe-LDH reduced the recombination of photogenerated electrons and holes (Figure 5a). At the same time, Fe species captured photogenerated holes and served as active sites for oxygen evolution reaction. For TiO_2_/ZnFe-LDH-E, the light radiation resulted in the stronger interaction between Zn 2p_3/2_ and Ti 2p_3/2_, resulting in the enhanced separation and transfer efficiency of photogenerated charges (Figure 5b,c). Carbon nanodots (CDs) have superior rapid charge separation due to their unique structure [73]. Lv et al. [74] reported the introduction of CDs further improved carrier mobility and reduced overpotential for oxygen evolution of CDs/NiFe-LDH/BiVO_4_ photoanode, leading to enhanced water splitting ability. Yang et al. [75] constructed a novel CoFe-LDH/NiFe-LDH core-shell architecture supported on nickel foam by a hydrothermal and electrodeposition strategy. The heterostructure showed the lowest Tafel slope of 88.88 mV dec^−1^, indicating excellent HER kinetics. The outstanding kinetics of the HER reaction was attributed to the strong synergistic effect as well as the typical 3D interconnected architectures. The HER activity of the core-shell architecture electrode is similar to or better than many state-of-the-art HER electrocatalysts. Zhang et al. [76] fabricated hierarchical NiFe-LDH@NiCoP nanowires on nickel foam as electrodes by a hydrothermal–phosphorization–hydrothermal strategy. The 3D heterostructure NiFe-LDH@NiCoP/NF electrodes require a low overpotential of 120 and 220 mV to deliver 10 mA cm^−2^ for the HER and OER, respectively. The overall water splitting of the heterostructure electrodes showed a cell voltage of 1.57 V at 10 mA cm^−2^ and excellent stability. Due to the strong electronic interaction between the NiFe-LDH and NiCoP, the synthetic strategy and interface engineering of the heterostructure facilitated charge transfer and improved reaction kinetics.

**Figure 6:**
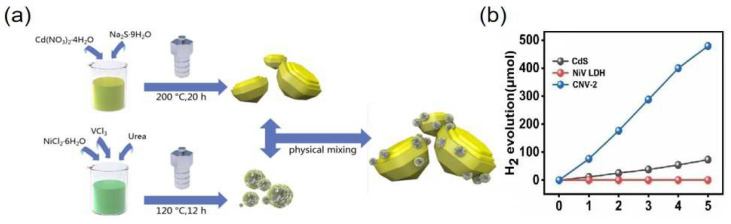
(**a**) The synthesis process of NiV-LDH/CdS. (**b**) The amount of hydrogen produced by CdS, NiV-LDH, and CNV-2. Copyright 2021 Elsevier [77].

The formation of positive–negative (PN) junctions is a common and effective method to improve photochemical water splitting performance. Yang et al. [77] used NiV-LDH and CdS to form P-N heterojunctions by physically mixing them together in a mass ratio of 1:10 (Figure 6a). The formed NiV-LDH/CdS heterostructure had excellent electron-hole separation ability, and the hydrogen evolution efficiency is significantly greater than that of pure NiV-LDH and CdS (Figure 6b). Sahoo et al. [78] constructed a heterojunction between Co(OH)_2_ and ZnCr-LDH by an ultrasonication method. The H_2_ and O_2_ evolution apparent conversion of optimized Co(OH)_2_-modified ZnCr LDH sample reached 13.12% and 6.25% in 2 h, respectively.

### 3.2. CO_2_ Reduction

Currently, the greatest threat to ecosystems is climate change. In order to achieve the plan specified in Conference of the Parties 21, energy and industrial processes need to reduce carbon emissions by 60% to limit global temperature rise to 2 °C [79]. There are also a number of ways to reduce the environmental impact of CO_2_: carbon capture and storage (ccs) chemical cycle capture, thermal decarbonization, photo(-electro)chemical reduction, and so on [80]. While reducing CO_2_, it is highly anticipated that CO_2_ can be used to generate electricity and convert it into more valuable compounds [81,82]. However, the traditional CO_2_ absorption method requires high temperature and pressure. A fresh LDH is not able to capture CO_2_, but LDHs forming a metal oxide mixture will have the ability to capture CO_2_ [83].

At present, the photochemical CO_2_ reduction attracts much attention due to the mild reaction condition. For photochemical CO_2_ reduction, when the energy of the absorbed light is greater than the band gap energy of LDHs, electron–hole pairs are produced. The photochemical CO_2_ reduction is roughly divided into three steps: (1) CO_2_ adsorption; hydroxyl groups adsorption on the surface [84], and interlayer anions adsorption [85]; (2) separation and migration of photogenerated charges [86]; (3) CO_2_ reduction reaction; CO_2_ will be reduced to hydrocarbons or CO by electrons [87]. The difference between PEC and photocatalytic (PC) CO_2_ reduction is that photoelectrocatalysis uses light and bias voltage to reduce CO_2_. Light performs as the drive, and the bias voltage improves the catalysis efficiency. Photosemiconductor structure, intrinsic properties, and active centers on the surface affect the efficiency of CO_2_ reduction in PEC [88].

**Figure 7:**
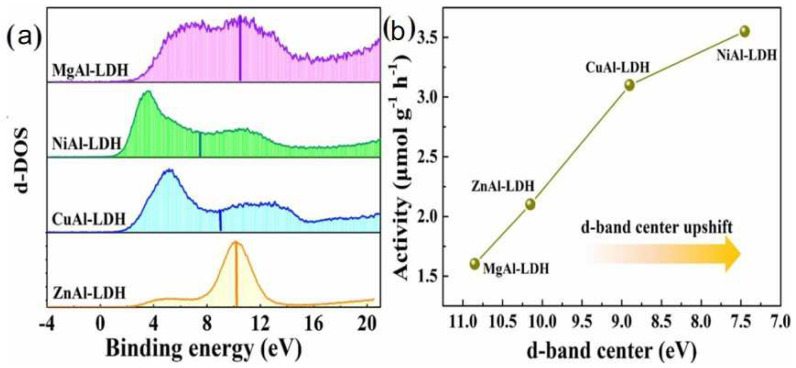
(**a**) Position of d−band center of the different LDHs. (**b**) Relationship of activity and the d−band center position for LDHs [89]. Copyright 2021 Elsevier.

In the photocatalytic CO_2_ reduction by LDH, the amount of CO_2_ absorbed depends on the type of divalent metal cation. Wang et al. [89] reported the bond strength between CO_2_ and MAl-LDH was relevant to the position of the d-band center. The higher the position of the d-band center, the higher the photocatalytic activity for CO_2_ reduction (Figure 7). The reduction capacity of CO_2_ was as follows: NiAl-LDHs > CuAl-LDHs > ZnAl-LDHs > MgAl-LDHs.

In photochemical reduction of CO_2_, the reduction of H_2_O tends to compete with the reduction of CO_2_ for electrons [90]. Therefore, much effort has been made to improve the selective reduction of CO_2_ by LDHs. Tan et al. [91] successfully obtained a composited photocatalyst with ruthenium and NiAl-LDH. The experimental results confirmed that a monolayer NiAl-LDH (m-NiAl-LDH) could completely suppress the hydrogen evolution reaction under a longer wavelength irradiation (λ > 600 nm). This phenomenon was attributed to the metal-induced defect states in the forbidden zone of m-NiAl-LDH. Photogenerated electrons only localized at the defect state, and the driving force of the defect state (0.313 eV) could reduce CO_2_ to CH_4_ instead of H_2_O reduction. Wang et al. [92] successfully prepared NiO samples with different vacancy amounts by calcinating NiAl-LDH. The vacancy concentrations of Ni and O determine the selectivity of CO_2_ reduction under visible light irradiation. The NiAl-275 sample with the highest defect concentration has the highest selectivity for CH_4_ (22.8%).

**Figure 8:**
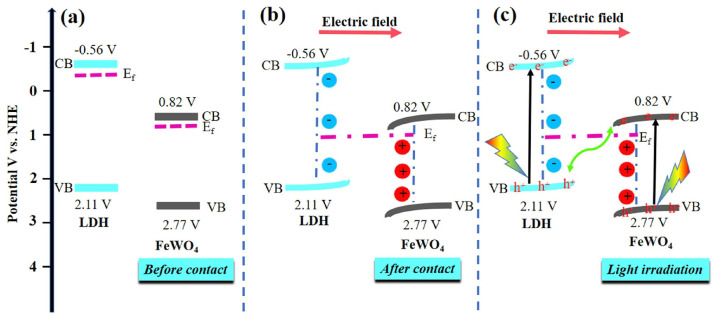
Schematic energy band diagrams of FeWO4 and NiAl−LDH (**a**) before and (**b**) after heterojunction formation. (**c**) Photogenerated charges transfer pathway in FeWO_4_/NiAl−LDH heterostructure under light irradiation [93]. Copyright 2021 Elsevier.

Constructing heterostructure still effectively improves the photo(electro)chemical performance of CO_2_ reduction. Lin et al. [93] prepared a FeWO_4_/NiAl-LDH(FWLDH) heterostructure using NiAl-LDH flower-like spheres and FeWO_4_ nanoflakes. The NiAl-LDH and FeWO_4_ formed a direct Z-scheme heterostructure. Tight binding of the heterostructure interface resulted in a larger specific surface area and thus formed more active sites. The internal electric field enhanced the separation and transport of photogenerated electrons, leading to the prominent improved photoelectron reduction ability of the NiAl-LDH (Figure 8). The photocatalytic CO yield of 10%FWLDH was 2.4 times than that of the original NiAl-LDH. Song et al. [94] fabricated a MgAl-LDO/carbon nitride with nitrogen defect (MgAl LDO/N_v_-CN) 2D heterostructure. The photocatalytic activity of 10% MgAl LDO/N_v_-CN for CO_2_ reduction was seven times than that of pure g-C_3_N_4_ under visible light illumination. Liu et al. [95] synthesized ultrathin Cu_2_O/CuCoCr-LDH p-n type heterojunction nanosheets (U-Cu_2_O/CuCoCr-LDH) as the cathodes of PEC. The photogenerated electrons at the photocathode reduced CO_2_ to CO and CH_4_. The maximum CO product yield of photoelectrocatalysis was 1167.6 mmol g^−1^h^−1^, which was approximately four times higher than that of electrocatalysis. The obvious improvement of photoelectrocatalytic performance was attributed to the internal electric field constructed by Cu_2_O and CuCr-LDH, which accelerates the separation of carriers.

LDHs have also been used in the electrocatalytic reduction of CO_2_. Fu et al. [96] prepared a monolayer NiFe-LDH catalyst using a solid-phase exfoliation method as an electrode for CO_2_ electroreduction. The optimized NiFe-CN-1 catalyst (NiFe-LDH was 1 wt%) exhibited a faradaic efficiency of CO generation of 93.5 % at 0.8v (vs. RHE). The excellent electrocatalytic performance originates from effective exposure of Ni and Fe active sites doped on the char material and efficient proton transfer channels of NiFe-LDH. Iwase et al. [97] prepared 2D CuAl-LDH as an electrocatalyst for electrochemical CO_2_ reduction (CO_2_RR). The optimized CuAl-LDH exhibited a faradaic efficiency of 42% for CO_2_ reduction to CO and 22% formate generation. It was found that the size of the LDH sheet was a key of CO_2_RR activity.

### 3.3. Contaminant Degradation

In 2015, about 9 million people died from environmental pollution, of which 1.8 million people died from diseases caused by water pollution [81]. Organic contaminants, as an important source of water pollution, are difficult to biodegrade because of their stability [98]. Photo(electro)catalysis has attracted extensive attention for solving environmental pollution problems, due to low cost, no pollution, and mild conditions [99,100].

Organic contaminant degradation by photo(electro)catalysis can be broadly divided into three steps: (1) The adsorption of the organic contaminant [101]. (2) The separation and transfer of photogenerated charges: This step is the key to improving photochemical activity [102]. (3) Redox reactions: Organic contaminant are converted to carbon dioxide, water, and inorganic acids by participating in redox reactions [103].

Recently, LDHs, particularly, transition metal-based LDHs (TLDHs), have emerged as promising candidates for contaminant degradation by photo(electro)catalysis [104]. Baliarsingh et al. [105] investigated the effect of M^2+^ (Co, Ni, Cu, and Zn) in M^II^/Cr-LDH to photodegrade methyl orange (MO). Among them, CoCr-LDH showed the highest photoactivity for MO (90% MO removal in 3 h). The improved photocatalytic activity of CoCr-LDH is mainly attributed to the excitation of M^2+^–O–Cr^3+^ bridge bonds under visible light irradiation and the effective transfer of photogenerated charge through the bridge bonds, which leads to the production of hydroxyl radicals and superoxide radicals. Zhao et al. [106] synthesized a series of MCr-LDH (M = Cu, Ni, Zn) samples with visible light response. The MCr-LDH samples have excellent photocatalytic activity for degradation of Sulforhodamine-B, Congo red, chlorinated phenol, and salicylic acid sodium. Experimental and computational results indicate that the obvious excellent visible light photocatalytic activity of MCr-NO_3_-LDHs is attributed to the low band gap and the abundant surface OH groups. The visible light response was induced by a d–d transition of CrO_6_ octahedra.

**Figure 9:**
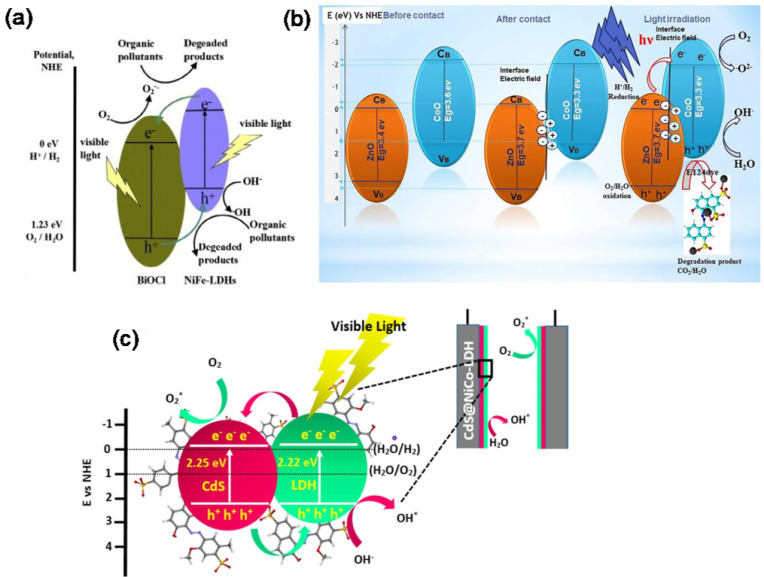
(**a**) The photocatalytic mechanism of BiOCl/NiFe−LDH heterostructure [108]. Copyright 2015 Elsevier. (**b**) The possible photodegradation mechanism of ZnO/CoO [109]. Copyright 2022 Elsevier. (**c**) Proposed photoelectrocatalytic degradation mechanism of CdS/NiCo−LDH heterojunctions [110]. Copyright 2022 Elsevier.

Construction of heterostructures is a common and effective approach to address the low photogenerated charge transport efficiency of LDHs. Megala et al. [107] obtained NiAl-LDH/CuWO_4_ heterostructures by a one-pot hydrothermal method. The photodegradation rate of LDH with 5% CuWO_4_ for methylene blue (MB) dye reached 87.5% in 5 h. The enhanced photocatalytic ability of NiAl-LDH/CuWO_4_ nanocomposite mainly originates from the heterojunction, which effectively promotes the separation of photogenerated charges. Ma et al. [108] synthesized BiOCl-NiFe-LDH composites using NiFe-Cl-LDH and Bi(NO_3_)_3_ as precursors. Photocatalytic activity of BiOCl-NiFe-LDH composites for Rhodamine B (RhB) degradation was 4.11 times higher than that of BiOCl. The heterostructure formed by BiOCl and NiFe-LDH can transfer photogenerated electron-holes in time (Figure 9a). At the same time, the highly dispersed BiOCl on the NiFe-LDH surface facilitates the formation of ·OH. Walaa R. Abd-Ellatif et al. [109] prepared ZnCo-LDH by a co-precipitation method and then obtained LDO (ZnO/CoO composite) by calcination. The ZnO/CoO composite formed S-scheme heterojunctions, as shown in Figure 9b. The removal rates of LDH calcinated at 300 °C for ponceau 4R (E124) and tartrazine (E102) were 90% and 80%, respectively. Pirkarami et al. [110] prepared CdS/NiCo-LDH heterojunctions by a hydrothermal method to construct photoelectrodes (Figure 9c). The degradation efficiency of Allura Red under an alkaline environment was over 90%. During the degradation process, the N = N of Allura Red would first break and it would eventually become H_2_O, NO_3_, NO_2_, CO_2_, SO_3_, Na^+^. Lu et al. [111] prepared Ni foam@ZnO@ZnFe-LDH photoelectrodes through electrodeposition of ZnO and hydrothermally grown ZnFe-LDH. Ni foam@ZnO@ZnFe-LDH acted as photoelectrodes in the PEC process and effectively removed Cr (VI) and Acid Red 1 by the synergistic effect of photoelectrocatalysis. Experimental results indicated that the 2D/2D core-shell heterojunction formed by ZnO and ZnFe-LDH not only narrowed the bandgap of ZnO and increased visible light absorption, but also promoted electron-hole separation. Argote-Fuentes et al. [112] synthesized activated MgAl-LDH through the co-precipitation method as heterogeneous catalysts for degradation of Congo red dye. In the photoelectrocatalysis process under 0.5v bias, the photoelectrocatalytic degradation rate of the MgAl-LDH/Cu electrode reached 95% and was the highest compared with other degradation processes. The synergistic effect of the Cu^2+^ ions induced by electric current and the photogenerated electrons suppressed the recombination of the electron–hole in the catalyst, resulting in excellent catalytic activity of Congo red degradation.

## 4. Conclusion and Outlook

LDHs are promising 2D photo(electro)catalysts with the advantages of low cost, tunable composition, unique thermal decomposition and memory properties, delaminated layer, and abundant surface hydroxyls. The compositional flexibility of LDHs can tune band structure, improve the absorption capacity and separation of charge carriers, and change the selectivity of the reaction. Calcined LDHs form in situ heterojunctions between the metal oxides, resulting in improved photo/electrocatalytic performance. Delamination and calcination of LDHs introduces more vacancy defects and specific surface areas, leading to an increased number of reactive sites. These insights into structure–activity relationships of LDHs provide a theoretical basis for function-oriented design of LDH-based photo(electro)catalytic materials.

Although a great deal of exciting research has appeared for improving the photo(electro)catalytic performance and fulfilling the practical applications, the two major drawbacks that need to be addressed for LDHs are the structural instability in a low pH environment and a low quantum efficiency induced by its low conductivity. The structure regulation of LDHs should be an ideal strategy to overcome the drawbacks. LDH is calcined at a certain temperature and then recovered by the memory effect, which improves its structural stability in an acidic environment and its photo(electro)catalytic performance. However, the corrosion resistance mechanism of calcined LDH has not yet been given a certain explanation. Taking advantage of the tunable composition characteristics of LDHs, the doping and defect introduction can effectively improve the conductivity of LDHs, resulting in enhanced quantum efficiency. How to precisely control the composition and structures of LDHs is still a huge challenge, such as precisely controlling the thickness of LDH and adjusting the ratio between metal cations by the electrodeposition method.

The structure–performance correlations of LDH-based photo(electro)catalytic materials needs to be more deeply understood to provide theoretical guidance for the design of efficient LDH photo(electro)catalysts. In order to better explore the structure–activity relationship of LDHs, advanced and effective characterization methods should be vigorously developed and applied. In situ characterization techniques would more precisely investigate structural changes of LDHs under reaction conditions. Transient spectroscopic techniques facilitate the research of photogenerated electron-hole separation and transfer dynamics and should be wildly utilized. In addition, theoretical simulations, especially density functional theory calculations, are powerful tools to study the relationship between the structure and properties of LDHs. The combination of advanced and valid characterization technologies and theoretical simulations is necessary to reveal complex charge dynamics, which supply a more detailed understanding of photo(electro)catalytic mechanisms. Further creative investigations will overcome the challenges in photochemistry of LDHs and will continue to advance the photo(electro)catalytic applications of LDHs.

## Abbreviations

LDH	Layered double hydroxide
RHE	Reversible hydrogen electrode
NHE	Normal hydrogen electrode
OER	Oxygen evolution reaction
HER	Hydrogen evolution reaction
PEC	Photoelectrocatalytic
VBM	Valence band maximum
LDO	Layered double oxide
PN	Positive–negative
NTAs	Nanotube arrays
NAs	Nanoarrays
CD	Carbon nanodot
Nv	Nitrogen defect
PE	Photoassisted electrodeposition method
MO	Methyl orange
